# Negative Emotions in Chinese Frontline Medical Staff During the Early Stage of the COVID-19 Epidemic: Status, Trend, and Influential Pathways Based on a National Investigation

**DOI:** 10.3389/fpsyt.2021.567446

**Published:** 2021-12-23

**Authors:** Xiaoxiao Sun, Fei Xie, Beijing Chen, Peixia Shi, Sitong Shen, Zhaohua Chen, Yuan Yuan, Mengjia Zhang, Xuemei Qin, Yingzhe Liu, Yuan Wang, Qin Dai

**Affiliations:** ^1^Department of Nursing Psychology, Army Medical University, Chongqing, China; ^2^The First Affiliated Hospital, Army Medical University, Chongqing, China; ^3^Hospital of the 81st Group Army PLA, Zhangjiakou, China; ^4^Teaching and Research Support Center, Army Medical University, Chongqing, China

**Keywords:** COVID-19, negative emotion, status, trend, influential pathway, Chinese frontline medical staff

## Abstract

**Objective:** The outbreak of coronavirus disease 2019 (COVID-19), declared as a major public health emergency, has had profound effects on public mental health especially emotional status. Due to professional requirements, medical staff are at a higher risk of infection, which might induce stronger negative emotions. This study aims to reveal the emotional status of Chinese frontline medical staff in the early epidemic period to better maintain their mental health, and provide adequate psychological support for them.

**Methods:** A national online survey was carried out in China at the early stage of the COVID-19 epidemic. In total, 3025 Chinese frontline medical staff took part in this investigation which utilized a general information questionnaire, the Emotion Regulation Questionnaire (ERQ), and the Berkeley Expressivity Questionnaire (BEQ).

**Results:** At the early stage of COVID-19, anxiety was the most common negative emotion of Chinese medical staff, followed by sadness, fear, and anger, mainly at a mild degree, which declined gradually over time. Nurses had the highest level of negative emotions compared with doctors and other healthcare workers. Women experienced more fear than men, younger and unmarried medical staff had more anxiety and fear compared with elders and married ones. Risk perception and emotional expressivity increased negative emotions, cognitive reappraisal reduced negative emotions, while negative emotions led to more avoidant behavior and more physical health disturbances, in which negative emotions mediated the effect of risk perception on avoidant behavior tendency in the model test.

**Conclusion:** Chinese frontline medical staff experienced a mild level of negative emotions at the early stage of COVID-19, which decreased gradually over time. The findings suggest that during the epidemic, nurses' mental health should be extensively attended to, as well as women, younger, and unmarried medical staff. To better ensure their mental health, reducing risk perception and improving cognitive reappraisal might be important, which are potentially valuable to form targeted psychological interventions and emotional guidance under crisis in the future.

## Introduction

Coronavirus disease 2019 (COVID-19), similar to SARS in 2003, has been declared as a major public health emergency (1), and has had a profound influence on personal mental health (2, 3). Up to May 4, 2020, there were 3,349,786 confirmed cases and 238,628 deaths because of COVID-19 worldwide (4). Millions of medical staff worked on the frontline to fight against the disease, making them face a high risk of infection (5, 6) and huge mental pressure (7, 8). Thousands of Chinese medical personnel combated with COVID-19 on the frontline, of whom over 3,000 medical staff were infected with the virus as of February 20, 2020 (8). Therefore, the COVID-19 epidemic can be regarded as a crisis event for the general population especially for those frontline medical staff.

According to Myer and Conte's (9) triage assessment system (TAS) for crisis intervention, reactions to crisis events are divided into three domains: affective or emotional, behavioral, and cognitive. The TAS model offers us an understanding about the type of reactions people experience as well as the intensity of these reactions in crisis. TAS can also provide a quick, accurate, and easy-to-use method that is directly usable in the intervention process and can monitor individuals' progress during the crisis intervention process. Thus, the TAS model is not only a valuable tool in the assessment of individual reactions in crisis but also a guide in the identification of the complex interaction among the three domains (9).

The first and most significant response under crisis is emotional response. Fear, anxiety, anger, and sadness are universal negative emotions among medical staff when facing sudden and arduous public health issues (10, 11). Studies revealed (10) that fear was a prominent emotional response among healthcare workers, and 1.4 times that compared with non-clinical staff (12). Taking the SARS epidemic as an example, frontline medical personnel were more likely to experience stronger negative emotions, such as the fear of being infected themselves or infecting their family members and anxiety of uncertainty (10, 13, 14). Additionally, anxiety was another common negative emotion among medical personnel (15). Medical staff working in emergency, ICU, and respiratory departments were two times more likely to suffer from anxiety (12). As to the gender difference, women had more anxiety compared with men (16). Being female, having frequent contact with patients, inadequate protective supplies, and being overloaded work were all related to the high level of anxiety (17, 18), which might contribute to emotional exhaustion (19). Besides, healthcare workers also experienced anger and sadness during SARS (13). A variety of factors might increase substantial psychological stress on medical staff (11, 13), such as shortage of protective supplies, direct contact with patients, and overloaded work, which might increase the infection rate of medical staff consequently (17, 20, 21). Although there exist studies on the negative emotions of medical staff during past epidemics, previous studies did not observe the status and sources of negative emotions of medical staff in detail, which is guaranteed to better maintain their mental health, as well as to provide adequate psychological support for them.

Nevertheless, different medical professionals may have different negative emotional responses. Studies reported (22) that nurses were more worried compared with doctors during the A/H1N1 influenza pandemic. The overall level of distress of nurses was significantly higher than that of other medical staff (23). During the COVID-19 epidemic, nurses reported experiencing more severe mental health symptoms than those of physicians (16). However, the emotional status and trend of negative emotions at the early stage of the epidemic were not systematically investigated, which was potentially important to develop more targeted psychological support toward different types of medical staff, since they were equally treated in the current psychological intervention (23).

In terms of emotional states, personal emotions fluctuate instead of sustaining (24). As Gross (24) pointed out, an individual's emotions unfold over seconds to minutes, which suggests the dynamic changes of emotions as time goes by. The latest cross-sectional survey (12) on the COVID-19 epidemic investigating 2042 medical staff from Fujian Provincial Hospital in China revealed that about 70.6% of the medical staff suffered from a moderate to severe level of fear, and 22.6% had a mild to moderate level of anxiety. However, this investigation was carried out at very early stage of this epidemic (January 24, 2020), and did not continue to monitor the emotion change of medical staff. The trend of negative emotions of Chinese medical staff is needed to explore timely and targeted psychological support in different periods of COVID-19. In previous epidemics, studies found (19, 25) that the fear and anxiety of medical staff appeared immediately and then decreased at the early stage of the epidemic, indicating that the early epidemic stage would be a critical period to monitor the negative emotional responses of frontline medical staff, as well as to provide timely psychological assistance. We thus supposed that negative emotions might be continuously decreasing from the initial phase of the epidemic.

So far, only the negative emotion reaction under crisis has been discussed, while according to Myer's TAS model, cognitive and behavioral reactions of frontline medical staff also need to be paid attention to. Facing the COVID-19 crisis event, a full understanding of the negative emotional responses and its influential factors from cognitive and behavioral domains might help to provide comprehensive information to formulate effective psychological interventions in future.

Studies reported (26) that risk perception in the cognitive domain might induce negative emotion. One study showed (27) that the level of fear elevated when people perceived a higher risk perception of SARS. Kushnir et al. (28) also reported that people tended to overestimate the risk of events that rarely happened while eliciting intense fears. Moreover, irrational risk perception would bring unnecessary anxiety and panic in turn (26). According to the Health Belief Model (29), risk perception could also influence individual behaviors. The high risk perception could affect medical staff's willingness to care for patients, especially when they were afraid of infecting their family members (30). Therefore, it could be assumed that the risk perception might influence the negative emotions and behavior of frontline medical staff under this epidemic.

Emotions might change behavior pattern. In terms of the relationship between negative emotion and behavioral reaction, studies revealed that fear was linked with avoidant behavior for protecting individuals from dangers (31, 32). For example, some medical staff were reluctant to work or determined to resign due to the fear of being infected or infecting their family members during the SARS epidemic (17, 33). The results suggested that negative emotions might promote the avoidant behavior tendency of medical personnel in the present epidemic, which need further evidence.

Stress would not only induce negative emotions, but also activate physical response. As mentioned above, the high level of negative emotion might cause physical function disturbance of frontline medical staff during an epidemic, such as headaches (7), anorexia (10), sleep disorders (34), and pain (35). Indeed, medical staff experienced more physical symptoms such as burnout, insomnia, and anorexia (10, 11, 13). However, the influence of negative emotions on the physical health of medical staff remains unknown under COVID-19, and we supposed that focusing on the physical response of frontline medical staff could broaden Myer's TAS theoretical system and reflect individual response toward COVID-19 more systematically.

Moreover, emotion regulation defined as the attempt to affect the way that one experiences or expresses emotions (24), can be used to help frontline medical staff deal with emotion reactions under the COVID-19 crisis. Gross and John (36) proposed that cognitive reappraisal and expressive suppression were two common emotion regulation strategies. Cognitive reappraisal, defined as the attempt to reinterpret the situation eliciting the emotion in the manner of changing its emotional impact (37, 38), could reduce negative emotions as reported (37, 39). While expressive suppression is defined as the attempt to hide, suppress, or reduce the ongoing emotion-expressive behavior (40). Cognitive reappraisal is related to healthier emotion and better wellbeing compared with expressive suppression (41). Additionally, emotional expressivity, which means expressing emotion through verbal, non-verbal, and physiological channels (42), also has an influence on negative emotions besides cognitive reappraisal and expressive suppression. Gross also pointed out that emotional expressivity as an opposite regulation strategy of expressivity suppression, might have a unique influence on emotions, correlating with negative emotion and mental health problems (43). Importantly, a study showed (44) that only expressive suppression significantly modulated fear under an epidemic, while other emotional regulation strategies did not, which suggested that different types of emotional regulation had different effects on fear emotion. However, previous research usually observe the effect of emotion regulation strategies on emotion in total, while the effect of different types of emotional regulation strategies on different types of emotions under crisis was not systematically revealed.

In summary, our present study aims to investigate the emotional status of Chinese frontline medical staff at the early period of the COVID- 19 epidemic, and further explore its trend and influential pathway. Our hypotheses were:

1) Chinese frontline medical staff might have a certain degree of negative emotions at the early stage of the COVID- 19 epidemic;
2) Nurses might have a higher level of negative emotions compared with other medical professionals;
3) At the early stage of the COVID- 19 epidemic, negative emotions of the Chinese frontline medical staff might decrease significantly;
4) Risk perception and emotion regulation might influence negative emotion;
5) Negative emotion might have a potential effect on avoidant behavior tendency and physical health;
6) Negative emotion might have a mediation effect between risk perception and avoidant behavior tendency and physical health.

## Methods

### Participants

A cross-sectional online survey was used to assess the emotional responses of Chinese frontline medical staff with a convenience sampling method. Doctors, nurses, and other medical staff, who cared for COVID-19 patients in the designated hospitals during this epidemic were eligible for this online national investigation from January 27 to February 11, 2020. Participants answered the questionnaire through an online link based on their personal will. A total of 4,100 medical staff responded, and 3,025 questionnaires were completed, which were from all provinces in China. Incomplete and halfheartedly filled in questionnaires were excluded from formal analysis. As depicted in Supplementary Table 1, there were 1,916 (63.3%) women and 1,109 (36.7%) men, aged between 20 and 65 years old. Among them, 1,237 (40.9%) were doctors, 1,371 (45.3%) were nurses, and 417 (13.8%) were other medical staff. In addition, 1,852 (61.2%) were married, 1,067 (35.3%) were unmarried, and 106 (3.5%) were divorced or widowed. Over 96.6% of the participants had college and postgraduate or higher educational levels. The participants covered all provincial administrative regions in China, which were divided into six groups of provinces according to the number of confirmed cases (above 10,000, 1,000–9,999, 500–999, 100–499, 10–99, and 1–9).

### Instruments

General information: Basic information about demographic characteristics, including gender, age, degree of education, marital status, and city (number of confirmed cases above 10,000, 1,000–9,999, 500–999, 100–499, 10–99, and 1–9) were collected.

Negative emotions: To collect the degree of negative emotions, four questions were designed with five options (none, mild, moderate, severe, extremely severe/unbearable): How fearful (anxious, angry, sad) do you feel today? Exploratory factor analysis (EFA, principal axis factoring (PAF)) and reliability analysis were carried out and found that the KMO of this scale was 0.797, which accounted for as much as 64.931% of the total variance; the Cronbach's alpha was 0.82. Cognitive sources for negative emotions are listed in the Supplementary Materials.

Risk perception: To observe people's risk perception during the epidemic, three questions were designed (yes or no): “This is a severe outbreak,” “Epidemic is close to me,” “I am in danger.” Exploratory factor analysis [EFA, principal axis factoring (PAF)] and reliability analysis were carried out and found that the KMO of this scale was 0.622, which accounted for as much as 57.94% of the total variance; the Cronbach's alpha was 0.635.

Cognitive sources of public anxiety: To explore possible sources of public anxiety, 12 questions were investigated (yes or no): Do you feel anxious about the new confirmed cases, possible infection without isolation, death number, shortage of protective supplies, new suspected cases, possible infection without protection, new foci, insufficient cooperation of patients, insufficient protection, being isolated due to the epidemic, insufficient duty of medical staff, and others?

Cognitive sources of public anger: To explore possible sources of public anger, 10 questions were surveyed (yes or no): Do you feel anger about irresponsible rumors, possible infection without isolation, possible infection without protection, insufficient cooperation of patients, shortage of protective supplies, insufficient attention of unit, insufficient duty of medical staff, unsupported by families, being isolated due to the epidemic, and others?

Cognitive sources of public sadness: To explore possible sources of public sadness, eight questions were investigated (yes or no): Do you feel sadness about innocent people, shortage of protective supplies, helpless patients, exhausted medical staff, being infected by the virus, being isolated due to the epidemic, unsupported by families, and others?

Cognitive sources of public fear: To explore possible sources of public fear, 14 questions were investigated (yes or no): Do you fear being infected by the virus, infection of families, possible infection without isolation, new confirmed cases, death after infection, shortage of protective supplies, possible infection without protection, death number, disrupted work or study after the epidemic, new foci, new suspected cases, insufficient cooperation of patients, being isolated due to the epidemic, and others?

Avoidant behavior tendency: To observe potential avoidant behavior tendency during the epidemic, three questions were designed (yes or no): “I am intending to run away if possible,” “To escape isolation, I might not go to hospital if I am a suspected case,” “To protect myself and families, I might quit the job if I am a medical staff member.” The KMO of this scale was 0.687, which accounted for as much as 77.88% of the total variance; the Cronbach's alpha was 0.857.

Disturbed physical function: To observe potential disturbed physical health during the epidemic, three questions were designed (yes or no): “Within the past week, I cannot keep my regular schedule as usual,” “Within the past week, I cannot eat as well as usual,” “Within the past week, I cannot sleep as well as usual.” The KMO of this scale was 0.602, which accounted for as much as 56.20% of the total variance; the Cronbach's alpha was 0.607.

Emotional regulation strategies: The Emotion Regulation Questionnaire (ERQ) with 10 items was used in this investigation, which was designed by Gross and John (36) and translated into Chinese in 2007 (45). High scores indicate higher cognitive reappraisal and expressive suppression, respectively. The Cronbach's alpha coefficient was 0.827 for cognitive reappraisal and 0.714 for expressive suppression in this study.

The Berkeley Expressivity Questionnaire (BEQ) (43) was also used to observe personal emotional expression, which is comprised of 16 items and 3 subscales: impulse strength, negative expressivity, and positive expressivity. The Cronbach's alpha coefficient was 0.834 in this study.

### Procedures

Questions were designed and edited as an online questionnaire, which was approved by the Human Research Ethics Committee of the Army Medical University of China and Wenjuanxing online platform (www.wjx.top), a platform providing functions equivalent to Amazon Mechanical Turk. From January 27 to February 11, 2020,participants answered the questionnaire through an online link with their personal cellphone or computer based on his/her individual will; there was no adverse consequence if they refused or did not have time to fill in the online questionnaire. Each person took about 10–15 min to complete the questionnaire after they signed the electronic version of the informed consent form. All participants could get a professional psychological consultation and aid *via* the hotline at the bottom of the electronic questionnaire.

### Statistics

An independent *t*-test and one way ANOVA were conducted to observe the demographic characteristics of negative emotions. Independent *t*-test analysis was also carried out to observe the influence of risk perception on negative emotions. The Chi-squared test was carried out to observe the effect of negative emotions on avoidant behavior tendency and disturbed physical function. Pearson correlation was carried out to observe the correlation between negative emotions, risk perception, emotional regulation, avoidant behavior, and disturbed physical function. A structural equation model was carried out with AMOS 24.0 to test the interaction between variables based on the TAS theory model.

## Results

### The Negative Emotional Status of Frontline Medical Staff

#### The Demographic Characteristics of Negative Emotions

To observe the negative emotional features of frontline medical staff, an independent two-sample *t*-test and one-way ANOVA analysis were conducted, which indicated (Supplementary Table 1) that women had a higher level of fear compared with men [*t*_(3,023)_ = −3.288, *p* = 0.001, Cohen's *d* = 0. 12]. Medical staff aged 40–49 years old had lower levels of anxiety [*F*_(3,3,021)_ = 5.026, *p* = 0.002, partial-eta^2^ = 0.005, and fear *F*_(3,3,021)_ = 3.417, *p* = 0.017, partial-eta^2^ = 0.003]. Medical staff with postgraduate degrees or higher reported lower fear levels [*F*_(3,3,021)_ = 2.763, *p* = 0.041, partial-eta^2^ = 0.003]. Unmarried medical staff reported higher levels of anxiety [*F*_(3,3,021)_ = 3.200, *p* = 0.022, partial-eta^2^ = 0.003] and fear [*F*_(3,3,021)_ = 3.564, *p* = 0.014,partial-eta^2^ = 0.004]. As expected, medical staff from the city (over 10,000 confirmed cases) had higher levels of anxiety [*F*_(3,3,021)_ = 2.440, *p* = 0.004, partial-eta^2^ = 0.032] and anger [*F*_(3,3,021)_ = 3.412, *p* = 0.004, partial-eta^2^ = 0.006].

#### The Negative Emotions of Different Medical Staff

**Figure 1:**
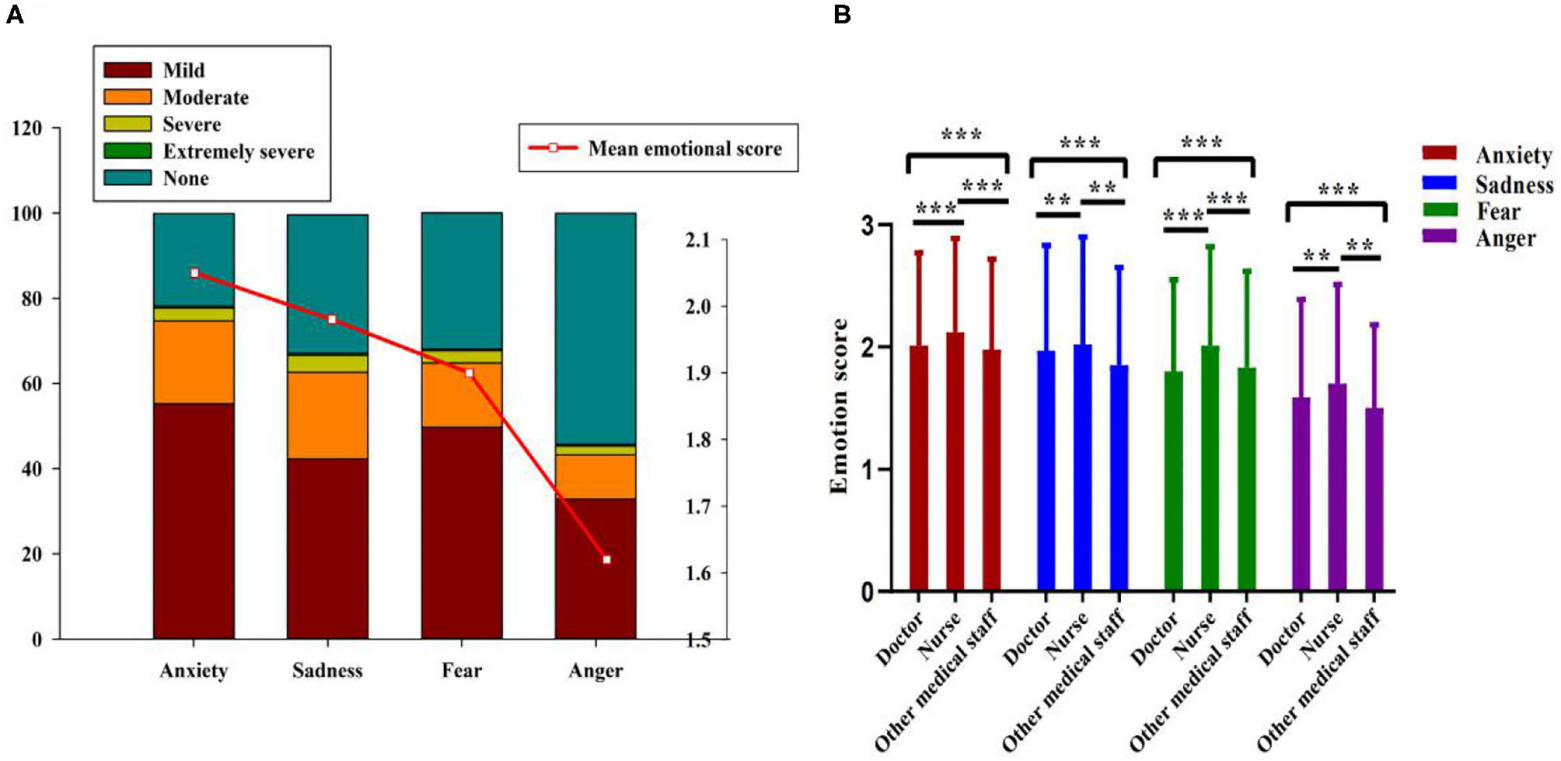
The negative emotional levels of different medical staff. **(A)** The overall negative emotional levels and response rates. **(B)** The negative emotional levels between different professionals. **p < 0.01, ***p < 0.001.

One way ANOVA (Figure 1A) showed that among the negative emotions, anxiety was the most prominent emotion in medical staff, followed by sadness, fear, and anger [*F*_(3,12,096)_ = 174.075, *p* < 0.001, partial-eta^2^ = 0.041]. The response selection rates of anxiety, sadness, fear, and anger were 78.3 67.5 68, and 45.8%. And 32.8–55.2% of medical staff rated their emotion at a mild degree. Further analysis (Supplementary Table 2) showed that compared with doctors and other medical staff, nurses had higher selection rates of anxiety [χ2_(2,3,025)_ = 16.776, *p* < 0.001], sadness [χ2_(2,3,025)_ = 11.908, *p* < 0.001], fear [χ2_(2,3,025)_ = 49.976, *p* < 0.001], and anger [χ2_(2,3,025)_ = 21.270, *p* < 0.001].

Figure 1B shows that nurses also reported higher levels of anxiety [*F*_(2,3,022)_ = 8.347, *p* < 0.001, partial-eta^2^= 0.005], sadness [*F*_(2,3,022)_ = 7.732, *p* < 0.001, partial-eta^2^ = 0.005], fear [*F*_(2,3,022)_ = 34.630, *p* < 0.001, partial-eta^2^ = 0.022], and anger [*F*_(2,3,021)_ = 9.719, *p* < 0.001, partial-eta^2^ = 0.006]. The results indicated that nurses experienced stronger negative emotions compared with other medical staff.

#### The Trend of Negative Emotions Over Time

**Figure 2:**
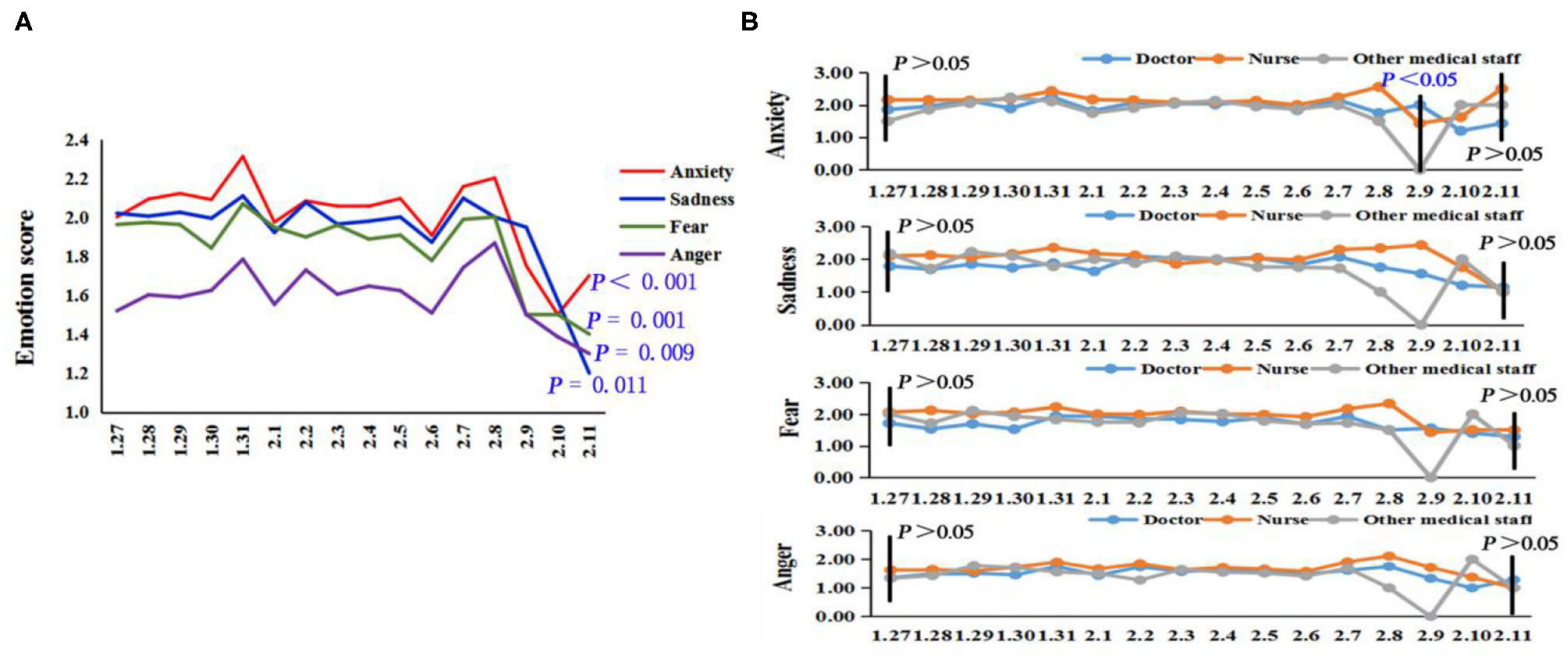
The trend of negative emotions over time. **(A)** The overall emotional trend of frontline medical staff. **(B)** The emotional trend of different medical professionals.

Figure 2A shows that the levels of negative emotions gradually declined over time during the early stage of the epidemic [anxiety: *F*_(15,3,009)_ = 3. 199, *p* < 0.001, partial-eta^2^ = 0.016, sadness: *F*_(15,3,009)_ = 2.016, *p* = 0.011, partial-eta^2^ = 0.010, fear: *F*_(15,3,009)_ = 2.577, *p* = 0.001, partial-eta^2^ = 0.013, and anger: *F*_(15,3,009)_ = 2.067, *p* = 0.009, partial-eta^2^ = 0.010]. Further analysis indicated (Figure 2B) that different medical professionals had a similar downward trend of sadness, fear, and anger over time without significant difference.

### Relationship Between Negative Emotions and Risk Factors, Emotional Regulation, Avoidant Behavior Tendency, and Disturbed Physical Function

#### The Sources of Negative Emotions

Supplementary Figure 1 shows that the shortage of protective supplies was the leading cause for all negative emotions. Specifically, the main sources for anxiety were possible infection without isolation, new confirmed cases, and possible infection without protection. The main sources for sadness were exhausted medical staff, helpless patients, and innocent people. The main sources for fear were possible infection without isolation, possible infection without protection, and being infected by the virus. The main sources for anger were possible infection without isolation, possible infection without protection, and irresponsible rumors.

The Chi-squared test was conducted to compare the percentage of sources between different medical professionals, which (Supplementary Table 3) indicated that compared with doctors, nurses had higher rates of “shortage of protective supplies” [χ2_(2,3025)_ =10.121, *p* = 0.006] and “new confirmed cases” [χ2_(2,3025)_ = 39.266, *p* < 0.001], which were sources of anxiety, as well as a higher percentage of “being infected by the virus” [χ2_(2,3025)_ =17.980, *p* < 0.001], which was a main source of fear.

#### The Impact of Risk Perception and Emotional Regulation Strategy on Negative Emotion

**Figure 3:**
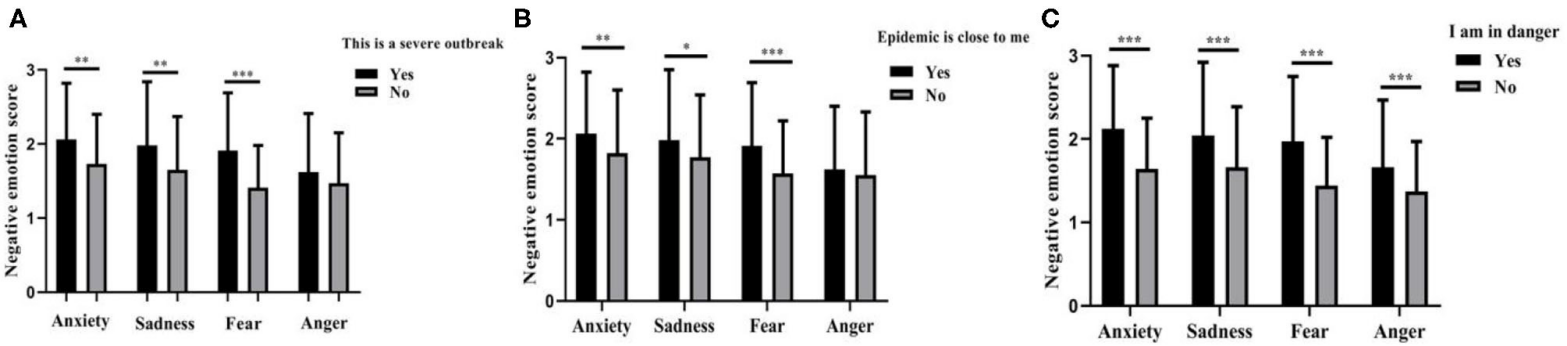
The impact of risk perception on negative emotions. **(A)** This is a severe outbreak. **(B)** The epidemic is close to me. **(C)** I am in danger. *p < 0.05, **p < 0.01, ***p < 0.001.

Figure 3 shows that medical staff answered “yes” for “this is a severe outbreak,” and had higher levels of anxiety [*t*_(3,023)_ = 2.980, *p* = 0.003, Cohen's *d* = 0.46], sadness [*t*_(3,023)_ = 2.677, *p* = 0.007, Cohen's *d* = 0.42], and fear [*t*_(3,023)_ = 4.455, *p* < 0.001, Cohen's *d* = 0.73] compared with those medical staff who had no risk perception. Similarly, medical staff answered “yes” for “the epidemic is close to me,” and reported higher levels of anxiety [*t*_(3,023)_ = 2.945, *p* = 0.003, Cohen's *d* = 0.31], sadness [*t*_(3,023)_ = 2.375, *p* = 0.018, Cohen's *d* = 0.26], and fear [*t*_(3,023)_ = 4.073, *p* < 0.001, Cohen's *d* = 0.47]. Moreover, medical staff answered “yes” for “I am in danger,” and reported higher levels of anxiety [*t*_(3,023)_ = 12.602, *p* < 0.001, Cohen's *d* = 0.70], sadness [*t*_(3,023)_ = 8.536, *p* < 0.001, Cohen's *d* = 0.47], fear [*t*_(3,023)_ = 13.567, *p* < 0.001, Cohen's *d* = 0.77], and anger [*t*_(3,023)_ = 7.061, *p* < 0.001, Cohen's *d* = 0.41].

Pearson correlation showed that (Table 1) cognitive reappraisal and expression suppression were negatively related to the levels of anxiety, fear, anger, and sadness (*r* = −0.049 ~ −0. 137, *p* < 0.01), while positive emotion expression, negative emotion expression, and impulse strength were positively correlated with negative emotions (*r* = 0.041 ~ 0. 166, *p* < 0.05).

**Table 1:** Correlation between negative emotion and emotion regulation strategy.

	**Cognitive reappraisal**	**Expressive suppression**	**Positive expressivity**	**Negative expressivity**	**Impulse strength**
Anxiety	−0.076**	−0.049**	0.078**	0.139**	0.142**
Anger	−0.137**	−0.059**	0.084**	0.094**	0.151**
Sadness	−0.057**	−0.026	0.069**	0.132**	0.124**
Fear	−0.094**	−0.051**	0.096**	0.166**	0.148**

** *p < 0.01*.

#### The Effects of Negative Emotions on Avoidant Behavior Tendency and Disturbed Physical Function

**Figure 4:**
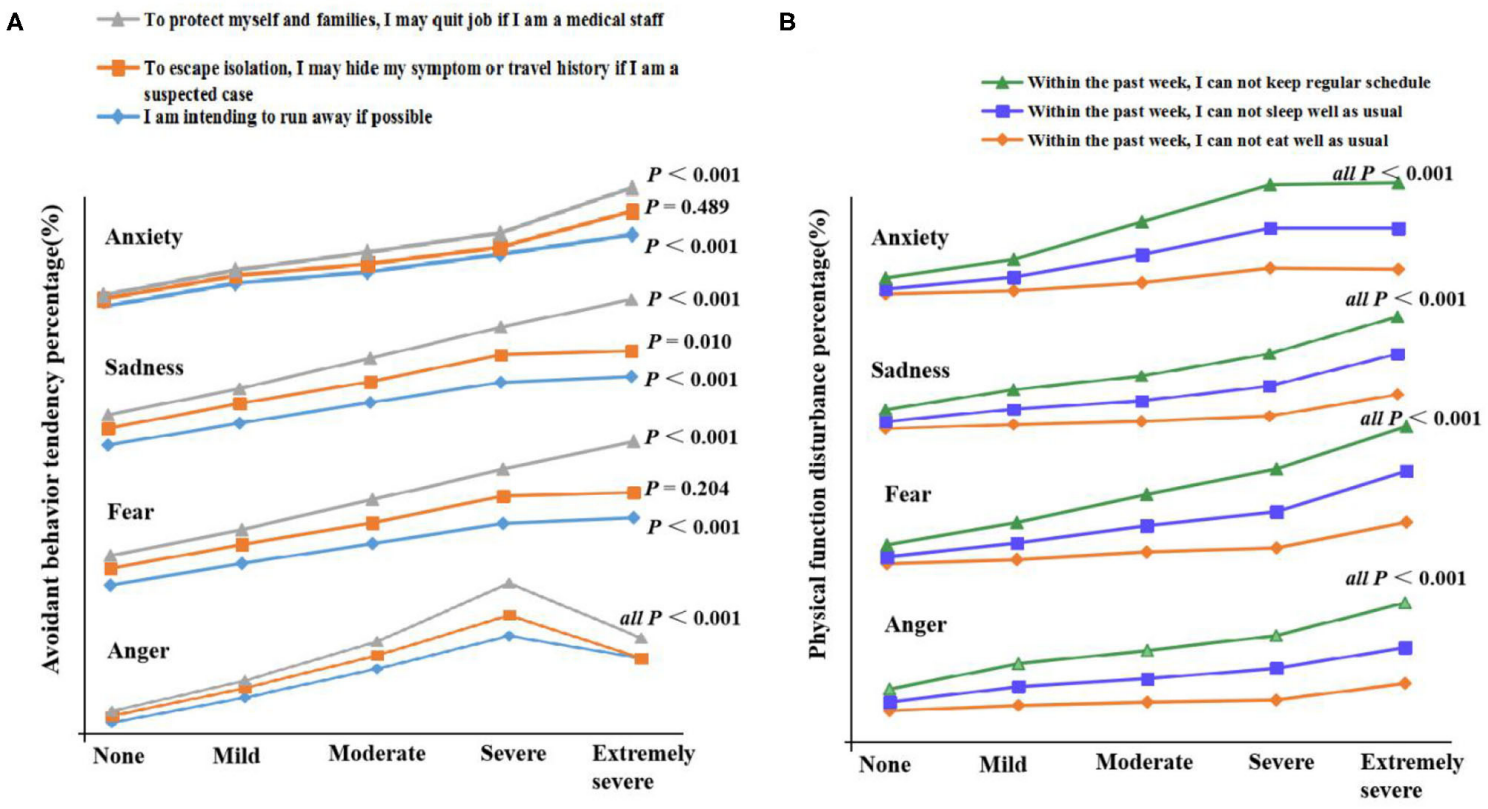
The effects of negative emotions on avoidant behavior tendency and disturbed physical function. **(A)** Avoidant behavior tendency. **(B)** Disturbed physical function.

Figure 4 indicates that negative emotions increased avoidant behavior tendency [χ2_(4,3,025)_ = 12.530 ~ 145.929, all *p* < 0.01] and disturbed physical function [χ2_(4,3,025)_ = 46.331 ~ 319.721, all *p* < 0.001] with increasing tendency as the enhancement of negative emotions (Supplementary Table 4).

### Emotional Models of Frontline Medical Staff

#### Confirmatory Factor Analysis (CFA) Results

Supplementary Figure 2 shows that the CFA analysis on risk perception, disturbed physical function, and avoidant behavior tendency indicated an ideal model fit (46) (χ*2* = 0, df = 0, NFI = 1.000, IFI = 1.000, CFI = 1.000, RMSEA = 0.012 ~ 0.052), whereas the CFA coefficients of negative emotions also showed a satisfied model fit (χ*2* = 4.998, df = 1, χ*2* /df = 4.998, NFI = 0.999, IFI = 0.999, CFI = 0.999, RMSEA = 0.005).

#### Interaction Pathway Between Negative Emotions and Their Influential Factors

To further explore the interaction between negative emotions and risk perception, emotional regulation, avoidant behavior tendency, and disturbed physical function, a hypothesis-driven model test was carried out (χ*2* = 57.986, df = 9, χ*2*/df = 6.443, RMSEA = 0.042, GFI = 0.995, AGFI = 0.983, NFI = 0.973, IFI = 0.977, TLI = 0.946, CFI = 0.977).

**Figure 5:**
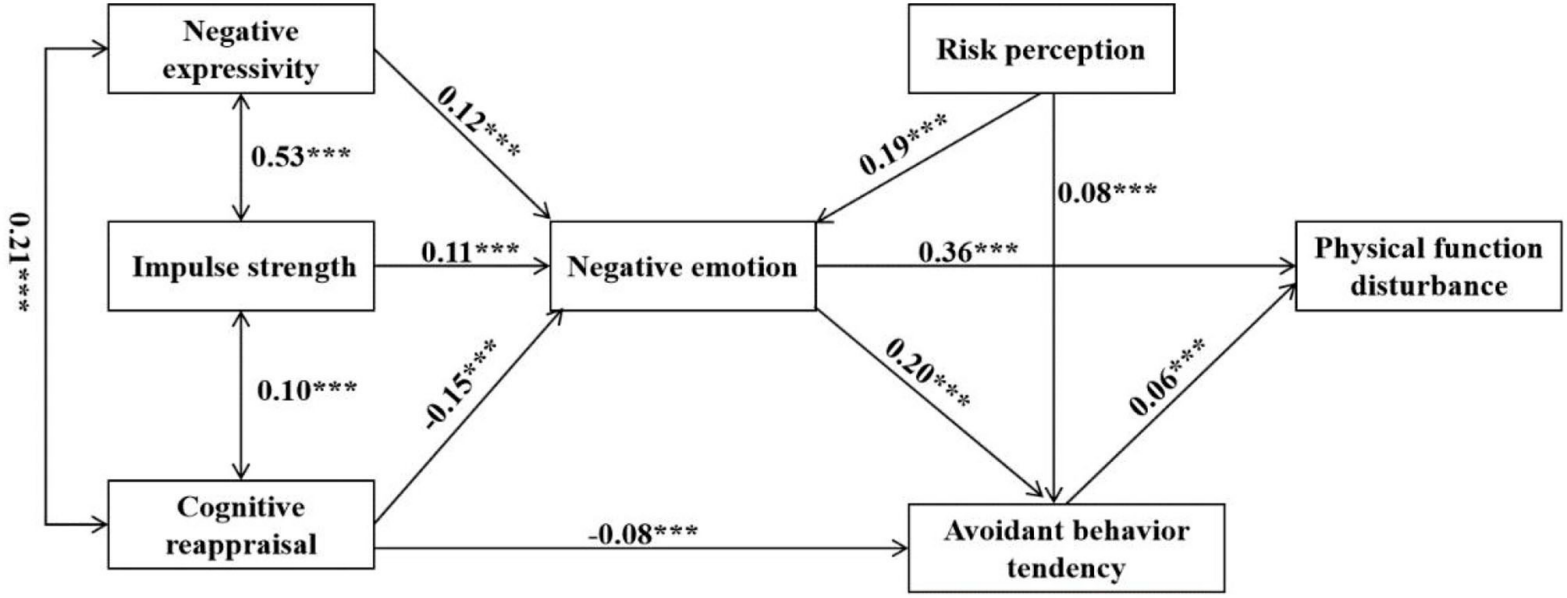
The influential pathway of the negative emotion of frontline medical staff. ***p < 0.001.

Figure 5 indicates that risk perception had a positive direct effect on negative emotion and avoidant behavior tendency, and an indirect effect on avoidant behavior tendency (0.029–0.048). Cognitive reappraisal had a negative effect while emotional expressivity had a positive effect on negative emotion. Negative emotion had a positive direct effect on avoidant behavior tendency and disturbed physical function, and an indirect effect on disturbed physical function (0.005–0.02). The results indicated a positive effect between negative emotion and risk perception, avoidant behavior tendency, and disturbed physical function, in which negative emotion and avoidant behavior tendency played a mediation role.

Moreover, cognitive reappraisal reduced negative emotion (β = −0.15, *p* < 0.001), and avoidant behavior tendency (β = −0.08, *p* < 0.001), while impulse strength and negative expressivity increased negative emotion (β = 0.11, 0.12, *p* < 0.001).

Multi-group analysis was conducted to verify the differences of models in three groups (doctors, nurses, and other medical staff). The result showed that there was no statistical difference in group comparisons (*p* = 0.367), which indicated that the different roles of medical staff did not affect this model.

## Discussion

In the current study, we explored the status, trend, and influential pattern of negative emotions in the Chinese frontline medical staff at the early stage of the COVID-19 epidemic *via* a nationwide investigation. Overall, we found that Chinese frontline medical staff experienced a mild level of negative emotions (i.e., fear, anxiety, anger, and sadness) in the early period of COVID-19, the trend of which decreased gradually over time. Additionally, nurses experienced a higher level of negative emotions, as well as women, younger, and unmarried medical staff. Risk perception and emotional expressivity increased negative emotions, cognitive reappraisal and expressive suppression reduced negative emotion, while negative emotions led to more avoidant behavior and more physical health disturbances, in which negative emotions mediated the effect of risk perception on avoidant behavior tendency and physical health disturbance of Chinese frontline medical staff.

### The Emotional Status of Frontline Medical Staff

The study revealed that Chinese frontline medical staff did experience different negative emotions, such as anxiety, sadness, fear, and anger. Among all the negative emotions, anxiety was the most common one for Chinese medical staff at the early stage of COVID-19. At the initial stage of COVID-19, it was normal for the public including frontline medical staff to experience negative emotions with the increasing number of confirmed cases and deaths. Our finding was consistent with other studies (48, 49), which also revealed that medical staff experienced a higher level of anxiety during epidemics. For example, a multicenter survey (50) reported that the prevalence of anxiety was 44.7% in 1,563 Chinese medical workers fighting against COVID-19. Another study (20) also showed that frontline health professionals were highly vulnerable to experiencing physical exhaustion, fear, and emotion disturbance. Notably, in this study, four negative emotions were measured based on the TAS theory, while previous studies (5, 47) rarely investigated all the four negative emotions in one research. Thus, the findings suggested that psychological interventions and support should focus on relieving these primary negative emotions under crisis for frontline medical staff.

As to the degree of those negative emotions, medical staff only suffered from a mild degree even in the toughest period of fighting COVID-19, while the degree of negative emotions was rarely reported in other research. This might be due to the fact that Chinese medical staff had experienced the SARS epidemic in 2003 and they had been well prepared for the challenge of future epidemics ever since.

Among all the frontline medical staff, nurses reported the highest level of negative emotions compared with other medical professionals in our investigation, which was consistent with previous studies (22, 23). Nurses were more worried than doctors during the A/H1N1 influenza pandemic (22), and the overall level of distress was significantly higher than that of other medical staff during SARS (23). Nurses were regarded as the most vulnerable group among frontline medical staff due to their high exposure risk to the novel coronavirus compared with other medical professionals. For example, nurses were always at the frontline to screen suspected patients (51) and had close contacts with patients (52). Besides nurses, women, younger, and unmarried frontline medical staff also reported a higher level of negative emotions. Therefore, more attention should be paid to maintain their psychological health under the current epidemic, and it is essential to support different kinds of medical staff differently.

### The Trend of Negative Emotions Over Time

The trend of negative emotions of Chinese frontline medical staff declined gradually as time went by, which was consistent with our hypothesis and previous findings (19, 25). However, according to one study conducted in Hong Kong in June 2003, researchers reported a relatively low level of distress in medical staff at the first stage of the epidemic, and they assumed that the level would be higher at the peak of the epidemic (23). Our result was inconsistent with their assumption. Two possible explanations for this finding: Firstly, the Chinese government played an active and productive role in the response to fighting against the epidemic, which enhanced medical staff's confidence toward the epidemic, and reduced their negative emotions effectively. Secondly, Chinese mental healthcare professionals provided psychological support for frontline medical staff in a timely and convenient manner (23), which guaranteed their mood effectively. The results suggested that along with the powerful executions of the government and epidemic control in China, medical staff's negative emotion decreased in a sense, which confirmed the effect of national epidemic control on public emotion. The finding also showed that psychological experts could provide psychological support at the beginning of an epidemic, which would be an important preventive measure to deal with the mental health problems of frontline medical staff.

### The Sources of Negative Emotions of Frontline Medical Staff

In this study, we found that the shortage of personal protective equipment (PPE) was the leading source of all negative emotions (i.e., fear, anxiety, anger, and sadness) of frontline medical staff at the early stage of the COVID-19 epidemic, which was also consistent with previous findings (13). Without standardized PPE, frontline medical staff were exposed to the higher risk of being infected with the virus. Besides, the potential infected individuals without quarantine and the suspected cases without protection were the main causes of anxiety, fear, and anger of frontline medical staff in this study, which was not previously reported. The results indicated that the quarantine and protection of suspected patients should be implemented strictly and effectively to reduce medical staff's psychological stress. Moreover, being infected by COVID-19 was also a primary source of fear among frontline medical staff. Thus, self-protection is of vital significance and much attention should be paid to train medical staff on how to use PPE before contacting patients (50). Our findings also showed that frontline medical staff felt sad about the existence of exhausted medical staff and severely infected patients. Together, results suggested that to reduce negative emotions of medical staff effectively, strategies should ensure PPE and implement protective procedures strictly and enhance therapeutic efficacy so that it might be most effective, which offers valuable evidence for the government.

### The Influential Effects Between Negative Emotions and Risk Perception, Avoidant Behavior, Physical Dysfunction, and Emotion Regulation

The findings confirmed that risk perception induced negative emotions of frontline medical staff significantly, which was consistent with the study (27) conducted during SARS. It showed that the level of fear elevated when people perceived a higher risk perception of the infectious disease (27). Therefore, reducing the risk perception of the epidemic may help healthcare workers reduce negative emotions. One systematic review of healthcare workers' perceptions of risk suggests that institutions need to ensure that appropriate infection control safeguards are in place to protect workers and their families (30). By doing so, negative emotions of medical staff are reduced effectively with lower risk perception.

Additionally, our findings showed the potential effect of negative emotion on personal avoidant behavior tendency, with the evidence of higher avoidant behavior tendency in medical staff with stronger negative emotion. Previous studies also found that some medical staff were reluctant to work or wanted to resign due to the fear of being infected or infecting their family members during the SARS epidemic (17, 33). The results suggested that reducing negative emotions may decrease the avoidant behavior tendency accordingly.

Besides, the findings also revealed the effect of negative emotion on personal disturbed physical function, with the evidence that a higher level of disturbed physical function in medical staff was related with a stronger level of negative emotion. The findings filled in the blank of previous empirical research between negative emotion and personal disturbed physical function, which also suggested that reducing negative emotions might decrease the physical function disturbance of frontline medical staff accordingly.

Moreover, the study innovatively verified the effect of emotion regulation strategy on negative emotion in the group of frontline medical staff during the early period of the COVID-19 epidemic. The findings showed that higher cognitive reappraisal and expressive suppression reduced the degree of negative emotions, while emotional expressivity increased negative emotions. The results indicate that the application of cognitive reappraisal and expressive suppression and less utilization of emotional expressivity may reduce negative emotions of frontline medical staff.

Together, risk perception induced negative emotions of frontline medical staff, emotion regulation strategies modulated negative emotions, and negative emotion had an effect on avoidant behavior tendency and disturbed physical function. The findings confirmed the TAS theory and further suggested the interaction between cognition, emotion, and behavior.

### The Pathway Between Negative Emotions and Their Influential Factors

The model test confirmed that risk perception increased negative emotion, which has been illustrated in a previous study (27). Moreover, emotional expressivity increased negative emotion, cognitive reappraisal and expressive suppression decreased negative emotion, and negative emotion increased disturbed physical health, which were first verified in this study. Among which, negative emotion played an important mediation role between risk perception and avoidant behavior and disturbed physical function. Therefore, measures to reduce risk perception of frontline medical staff should be taken in a timely manner to decrease negative emotion and avoidant behavior at the early period of the COVID-19 epidemic. For example, training courses about the disease and treatment might be an effective way to minimize risk perception (53, 54). Meanwhile, to better maintain normal psychological and physical function under crisis, intervention and guidance on fear emotion are critical. We also found that avoidant behavior could directly influence disturbed physical function, filling the gap of existing knowledge. Thus, we can reduce negative emotions and avoidant behavior of frontline medical staff to promote their physical function by adopting interventions like cognitive-behavioral therapy (CBT) (55, 56). This knowledge helps to reveal an influential pattern between negative emotion and risk perception, emotion regulation strategy, avoidant behavior tendency, and physical function.

Importantly, negative emotions could be reduced through cognitive reappraisal and reduced risk perception, followed by decreased avoidance behavior and physical impairment. The findings provide a guide for psychologists to promote crisis intervention in the group of frontline medical staff during the COVID-19 epidemic, which further broadens the application of the TAS model.

The strengths of our study are as follows: firstly, the current study was one of the earliest nationwide and large population-based online surveys targeted at Chinese frontline medical staff fighting against COVID-19 from January 27 to February 11, 2020, which covered all 32 provincial administrative regions of China. Secondly, our study was a continuous trend survey of negative emotions of frontline medical staff at the early COVID-19 stage. We also explored the emotion status of different medical professionals, which might be potentially important to draw up psychological interventions for the targeted population under a public health crisis. Thirdly, we also explored the influential effects between negative emotions and risk perception, avoidant behavior, physical dysfunction, and emotion regulation among Chinese frontline medical staff, which could help provide emotion support more precisely and scientifically in the future.

Several limitations also exist in the study. First, only one question was designed to evaluate each kind of the four common negative emotions, and the study lacked more details about negative emotions of Chinese frontline medical staff fighting. Second, the data of other influential factors of emotions, such as coping styles and resilience, were not collected in the present study, and thus future research can explore more variables interacting with the emotions of frontline medical staff during epidemics. Moreover, despite the fact that numerous statistically significant results were found in the study, the effect sizes were rather weak. Some bias might exist due to the online data collection though it was a very convenient and effective way to do the online survey during COVID-19.

## Conclusion

In conclusion, Chinese frontline medical staff experience a mild level of negative emotions (i.e., fear, anxiety, anger, and sadness) at the early stage of COVID-19, which decreases gradually over time. Nurses report a higher level of negative emotions, as well as women, younger, and unmarried healthcare professionals. To better ensure the mental health of medical staff, reducing risk perception and improving cognitive reappraisal might be important, which would be potentially valuable to form targeted psychological intervention and emotional guidance under crisis in the future.

## Data Availability Statement

The original contributions presented in the study are included in the article/Supplementary Material, further inquiries can be directed to the corresponding author.

## Ethics Statement

The studies involving human participants were reviewed and approved by the Human Research Ethics Committee of the Army Medical University of China. The patients/participants provided their written informed consent to participate in this study.

## Author Contributions

QD: research design, review, and supervision. XS: making questionnaires, data analysis, and writing original draft preparation. FX: review and editing. BC, PS, SS, ZC, YY, MZ, XQ, YL, and YW: data collection. All authors have read and agreed to the published version of the manuscript.

## Funding

QD claims that this study was supported by the Key Project of Natural Science Foundation of Chongqing (cstc2020jcyj-zdxmX0009), the Medical Innovation Project of Army Medical University (2019ZLX003), and the Key Project and Innovation Project of People's Liberation Army of China (18CXZ005).

## Conflict of Interest

The authors declare that the research was conducted in the absence of any commercial or financial relationships that could be construed as a potential conflict of interest.

## Publisher's Note

All claims expressed in this article are solely those of the authors and do not necessarily represent those of their affiliated organizations, or those of the publisher, the editors and the reviewers. Any product that may be evaluated in this article, or claim that may be made by its manufacturer, is not guaranteed or endorsed by the publisher.
