# Supplementary material for: Negative Emotions in Chinese Frontline Medical Staff During the Early Stage of the COVID-19 Epidemic: Status, Trend, and Influential Pathways Based on a National Investigation
Source: Front Psychiatry. 2021 Dec 23;12:567446. doi: 10.3389/fpsyt.2021.567446 (PMC8732867; doi:10.3389/fpsyt.2021.567446)
Supplement: Supplementary file 1 [file Data_Sheet_1.doc]

**Supplementary material**

**Table S1** Comparison of negative emotion scores under demographic variables and risk perception

| **Variables** | **Overall N(%)** | **Anxiety** | **Sadness** | **Fear** | **Anger** |
| --- | --- | --- | --- | --- | --- |
| **Total** | 3025 |  |  |  |  |
| **Gender** |  |  |  |  |  |
| Male | 1109(36.7) | 2.02(0.78) | 1.95(0.86) | 1.84(0.77) | 1.60(0.77) |
| Female | 1916(63.3) | 2.07(0.75) | 2.00(0.87) | 1.93(0.78) | 1.63(0.80) |
| ***t*** (Cohen's d) |  | -1.666(-0.07 ) | -1.733(-0.06) | -3.288**(-0.12) | -1.087(-0.04 ) |
| **Age(years)** |  |  |  |  |  |
| 20-29 | 704(23.3) | 2.08(0.75) | 1.97(0.86) | 1.94(0.79) | 1.62(0.75) |
| 30-39 | 1125(37.2) | 2.10(0.75) | 2.00(0.88) | 1.93(0.80) | 1.67(0.82) |
| 40-49 | 823(27.2) | 1.97(0.75) | 1.96(0.83) | 1.83(0.74) | 1.57(0.77) |
| 50-65 | 373(12.3) | 2.04(0.78) | 1.98(0.91) | 1.89(0.78) | 1.57(0.81) |
| ***F* (**partial-eta2**)** |  | 5.026**(0.005) | 0.358(0.000) | 3.417*(0.003) | 2.942(0.003) |
| **Educational level** |  |  |  |  |  |
| College | 2531(83.7) | 2.05(0.76) | 1.99(0.86) | 1.91(0.78) | 1.61(0.78) |
| Postgraduate or higher | 390(12.9) | 2.05(0.75) | 1.93(0.88) | 1.82(0.71) | 1.67(0.82) |
| ***t*** (Cohen's d) |  | 0.069(0.00) | 1.274(0.07) | 2.251*(0.12) | -1.425(-0.07) |
| **Marital status** |  |  |  |  |  |
| Married | 1852(61.2) | 2.03(0.76) | 1.97(0.87) | 1.87(0.79) | 1.60(0.78) |
| Unmarried | 1067(35.3) | 2.10(0.74) | 2.00(0.87) | 1.95(0.77) | 1.63(0.79) |
| Divorced | 88(2.90) | 1.91(0.71) | 2.03(0.76) | 1.78(0.69) | 1.73(0.85) |
| Widowed | 18(0.60) | 2.17(1.04) | 2.11(1.18) | 2.17(0.99) | 1.78(1.00) |
| ***F* (**partial-eta2**)** |  | 3.200*(0.003) | 0.622(0.001) | 3.564*(0.004) | 1.233(0.001) |
| **Profession** |  |  |  |  |  |
| Doctor | 1237(40.9) | 2.01(0.77) | 1.94(0.87) | 1.78(0.74) | 1.58(0.79) |
| Nurse | 1371(45.3) | 2.12(0.76) | 2.05(0.88) | 2.02(0.79) | 1.68(0.80) |
| Other medical staff | 417(13.8) | 1.99(0.72) | 1.88(0.81) | 1.85(0.78) | 1.53(0.72) |
| ***F* (**partial-eta2**)** |  | 8.347***(0.005) | 7.732***(0.005) | 34.630***(0.022) | 8.949***(0.006) |
| **Confirmed cases in city or area** |  |  |  |  |  |
| ≥10000 | 137(4.52) | 2.15(0.86) | 2.07(0.93) | 1.98(0.88) | 1.79(0.94) |
| 1000-9999 | 579(19.1) | 2.00(0.74) | 1.92(0.82) | 1.86(0.74) | 1.53(0.73) |
| 500-999 | 1189(39.3) | 2.07(0.73) | 1.98(0.84) | 1.91(0.76) | 1.61(0.76) |
| 100-499 | 886(29.3) | 2.04(0.77) | 2.00(0.92) | 1.89(0.79) | 1.65(0.82) |
| 10-99 | 219(7.2) | 2.16(0.81) | 2.02(0.87) | 1.91(0.85) | 1.67(0.84) |
| 1-9 | 15(0.50) | 1.80(0.68) | 2.13(0.83) | 1.67(0.62) | 1.73(0.59) |
| ***F* (**partial-eta2**)** |  | 2.440*(0.004) | 1.142(0.002) | 0.990(0.002) | 3.412**(0.006) |
| “This is a severe outbreak” |  |  |  |  |  |
| Yes | 2976(98.4) | 2.06(0.76) | 1.98(0.86) | 1.91(0.78) | 1.62(0.79) |
| No | 49(1.6) | 1.73(0.67) | 1.65(0.72) | 1.41(0.57) | 1.47(0.68) |
| ***t*** (Cohen's d) |  | 2.980**(0.46) | 2.677**(0.42) | 4.455***(0.73) | 1.316(0.20) |
| “Epidemic is close to me” |  |  |  |  |  |
| Yes | 2934(97.0) | 2.06(0.76) | 1.98(0.87) | 1.91(0.78) | 1.62(0.78) |
| No | 91(3.0) | 1.82(0.78) | 1.77(0.77) | 1.57(0.65) | 1.55(0.78) |
| ***t*** (Cohen's d) |  | 2.945**(0.31) | 2.375*(0.26) | 4.073***(0.47) | 0.823(0.09) |
| “I am in danger” |  |  |  |  |  |
| Yes | 2591(85.7) | 2.12(0.76) | 2.04(0.88) | 1.97(0.78) | 1.66(0.81) |
| No | 434(14.3) | 1.64(0.61) | 1.66(0.73) | 1.44(0.58) | 1.37(0.60) |
| ***t*** (Cohen's d) |  | 12.602***(0.70) | 8.536***(0.47) | 13.567***(0.77) | 7.061***(0.41) |

**p* < 0.05, ***p* < 0.01, ****p* < 0.001

**Table S2** Comparison of emotional response rate between different professionals

| **N(%)** | **Total** | **Doctor** | **Nurse** | **Other medical staff** | ***χ2*** | ***p*-value** | **Effect size (Phi)** |
| --- | --- | --- | --- | --- | --- | --- | --- |
| Anxiety | 2369 (78.3) | 929 (75.1) | 1119 (81.6) | 321 (77.0) | 16.776 | < 0.001 | 0.074 |
| Sadness | 2041 (67.5) | 805 (65.1) | 969 (70.7 ) | 267 (64.0) | 11.908 | 0.003 | 0.063 |
| Fear | 2057 (68.0) | 764 (61.8) | 1021 ( 74.5) | 272 (65.2) | 49.976 | < 0.001 | 0.129 |
| Anger | 1384 (45.8) | 522 (42.2) | 690 (50.3 ) | 172 (41.2) | 21.270 | < 0.001 | 0.084 |

**Table S3** Comparison of primary sources of negative emotions between different professionals

| ***N*(%)** | **Doctor (D)** | **Nurse (N)** | **Other medical staff (OS)** | ***χ2*** | ***p*-value** | **Effect size**  **(Phi)** |
| --- | --- | --- | --- | --- | --- | --- |
| 1.Shortage of protective supplies (anxiety) | 972 (78.6) | 1140 (83.2) | 347 (83.2) | 10.121 | **0.006** | 0.058 |
| 2.Possible infection without isolation (anxiety) | 902 (72.9) | 1016 (74.1) | 301 (72.2) | 0.810 | 0.667 | 0.016 |
| 3.New confirmed cases (anxiety) | 755 (61.0) | 995 (72.6) | 281 (67.4) | 39.266 | **< 0.001** | 0.114 |
| 4. Possible infection without protection (anxiety) | 802 (64.8) | 904 (65.9) | 271 (65.0) | 0.378 | 0.828 | 0.011 |
| 5.Shortage of protective supplies  (sadness) | 957(77.4) | 1097(80.0) | 328(78.7) | 2.730 | 0.255 | 0.030 |
| 6.Exhausted medical staff (sadness) | 895(72.4) | 983(71.7) | 305(73.1) | 0.367 | 0.832 | 0.011 |
| 7.Helpless patients (sadness) | 579(46.8) | 669(48.8) | 205(49.2) | 1.278 | 0.528 | 0.021 |
| 8.Innocent people (sadness) | 573(46.3) | 660(48.1) | 203(48.7) | 1.146 | 0.564 | 0.019 |
| 9.Shortage of protective supplies (fear) | 806(65.2) | 927(67.6) | 289(69.3) | 3.094 | 0.213 | 0.032 |
| 10. Possible infection without isolation (fear) | 746(60.3) | 827(60.3) | 247(59.2) | 0.176 | 0.916 | 0.008 |
| 11. Possible infection without protection (fear) | 675(54.6) | 766(55.9) | 242(58.0) | 1.574 | 0.455 | 0.023 |
| 12.Been infected by virus (fear) | 544(44.0) | 715(52.2) | 193(46.3) | 17.980 | **< 0.001** | 0.077 |
| 13.Shortage of protective supplies (anger) | 878(71.0) | 1015(74.0) | 294(70.5) | 3.808 | 0.149 | 0.035 |
| 14. Possible infection without isolation (anger) | 842(68.1) | 953(69.5) | 298(71.5) | 1.808 | 0.405 | 0.024 |
| 15. Possible infection without protection (anger) | 776(62.7) | 883(64.4) | 272(65.2) | 1.195 | 0.550 | 0.020 |
| 16.Irresponsible rumor (anger) | 800(64.7) | 873(63.7) | 278(66.7) | 1.277 | 0.528 | 0.021 |
|  |  |  |  |  |  |  |
| ***Post hoc* analysis** | ***p*-value^＃^** | | ***p*-value^＃^** | | ***p*-value^＃^** | |
| 1.Shortage of protective supplies (anxiety) | D vs. N | **0.003** | D vs. OS | 0.042 |  |  |
| 3.New confirmed cases (anxiety) | D vs. N | **< 0.001** | D vs. OS | 0.020 | N vs. OS | 0.040 |
| 12.Been infected by virus (fear) | D vs. N | **< 0.001** |  |  |  |  |

**^＃^***Chi-squared test with the Bonferroni correction for multiple comparisons (p < 0.0167).*

**Table S4** The correlation between avoidant behavior and disturbed physical function with different degrees of negative emotions

|  | **Anxiety(%)** | | | | | | | | **Sadness(%)** | | | | | | | |
| --- | --- | --- | --- | --- | --- | --- | --- | --- | --- | --- | --- | --- | --- | --- | --- | --- |
|  | **None** | **Mild** | **Moderate** | **Severe** | **Extremely severe** | ***χ2*** | ***p*-value** | **Effect size**  **(Phi)** | **None** | **Mild** | **Moderate** | **Severe** | **Extremely severe** | ***χ2*** | ***p*-value** | **Effect size**  **(Phi)** |
| Avoidant behavior 1 | 6.6 | 20.8 | 27.6 | 38.3 | 50.0 | 124.730 | **< 0.001** | 0.203 | 22.0 | 15.3 | 25.9 | 42.6 | 35.7 | 70.337 | **< 0.001** | 0.152 |
| Avoidant behavior 2 | 4.7 | 4.6 | 4.9 | 4.3 | 14.3 | 3.057 | 0.489 | 0.032 | 6.1 | 3.7 | 5.2 | 9.6 | 7.1 | 12.530 | 0.010 | 0.064 |
| Avoidant behavior 3 | 2.4 | 3.4 | 7.1 | 8.5 | 14.3 | 28.909 | **< 0.001** | 0.098 | 4.9 | 2.8 | 5.9 | 9.6 | 14.3 | 24.554 | **< 0.001** | 0.090 |
| Physical function1 | 4.1 | 9.2 | 21.8 | 44.7 | 7.1 | 204.839 | **< 0.001** | 0.260 | 7.9 | 9.2 | 18.1 | 37.2 | 71.4 | 148.570 | **< 0.001** | 0.222 |
| Physical function2 | 7.8 | 21.3 | 44.2 | 62.8 | 64.3 | 319.721 | **< 0.001** | 0.325 | 17.4 | 19.9 | 35.7 | 72.3 | 78.6 | 216.755 | **< 0.001** | 0.268 |
| Physical function3 | 14.3 | 21.3 | 38.9 | 50.0 | 7.1 | 149.497 | **< 0.001** | 0.222 | 23.0 | 19.2 | 34.0 | 54.3 | 64.3 | 112.523 | **< 0.001** | 0.193 |
|  | **Fear (%)** | | | | | | | | **Anger (%)** | | | | | | | |
|  | **None** | **Mild** | **Moderate** | **Severe** | **Extremely severe** | ***χ2*** | ***p*-value** | **Effect size**  **(Phi)** | **None** | **Mild** | **Moderate** | **Severe** | **Extremely severe** | ***χ2*** | ***p*-value** | **Effect size**  **(Phi)** |
| Avoidant behavior 1 | 12.2 | 18.6 | 26.7 | 47.9 | 28.6 | 90.940 | **< 0.001** | 0.173 | 35.4 | 14.4 | 15.7 | 27.7 | 42.9 | 145.929 | **< 0.001** | 0.220 |
| Avoidant behavior 2 | 4.9 | 4.3 | 5.1 | 9.6 | 0 | 6.668 | 0.204 | 0.047 | 9.9 | 3.1 | 3.2 | 5.3 | 7.1 | 52.352 | **< 0.001** | 0.132 |
| Avoidant behavior 3 | 3.4 | 3.4 | 5.2 | 14.9 | 7.1 | 33.458 | **< 0.001** | 0.105 | 6.4 | 2.8 | 4.4 | 7.4 | 14.3 | 22.376 | **< 0.001** | 0.086 |
| Physical function1 | 7.3 | 10.3 | 17.8 | 27.7 | 50.0 | 78.638 | **< 0.001** | 0.161 | 16.8 | 9.4 | 11.3 | 17.0 | 57.1 | 54.889 | **< 0.001** | 0.135 |
| Physical function2 | 15.5 | 23.3 | 31.6 | 53.2 | 57.1 | 96.326 | **< 0.001** | 0.178 | 36.9 | 19.2 | 21.5 | 37.2 | 78.6 | 113.348 | **< 0.001** | 0.194 |
| Physical function3 | 21.6 | 22.2 | 28.6 | 48.9 | 42.9 | 46.331 | **< 0.001** | 0.124 | 42.8 | 17.9 | 20.1 | 29.8 | 42.9 | 169.706 | **< 0.001** | 0.237 |

Note: **Avoidant behavior 1** was “I am intending to run away if possible ”, Avoidant behavior 2 was “To avoidant isolation, I may hide my symptom or travel history if I am a suspected case,” Avoidant behavior 3 was “To protect myself and families, I may quit job if I am a medical staff ”. **Physical function 1** was “ Within the past week, I can not eat well as usual,” physical function 2 was “Within the past week, I can not sleep well as usual,” and physical function 3 was “Within the past week, I can not keep regular schedule.”


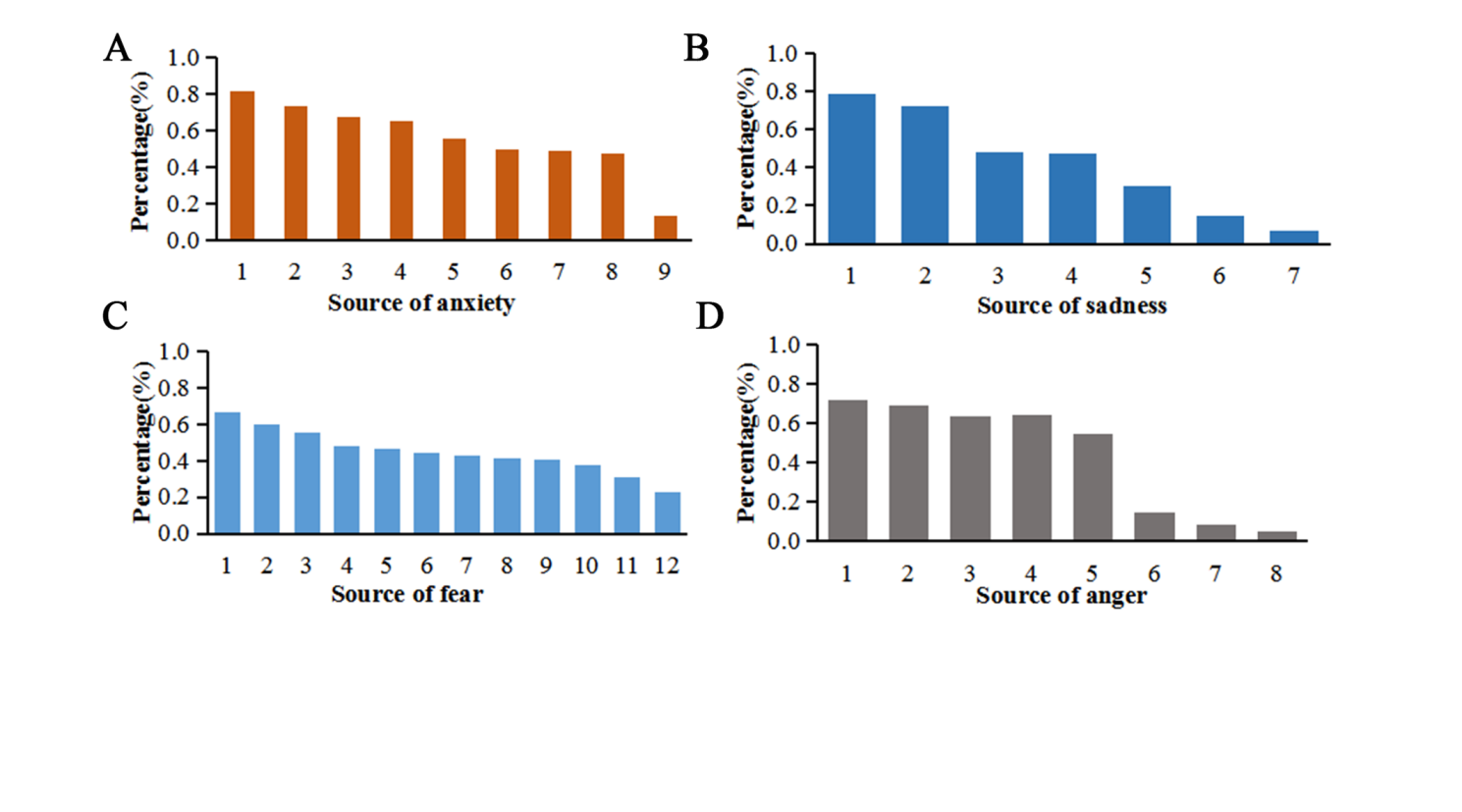


***A:*** *1= Shortage of protective supplies; 2= possible infection without isolation; 3= new confirmed cases;*

*4= possible infection without protection; 5= insufficient cooperation of patients; 6= new suspected cases; 7= death number; 8= new foci; 9= been isolated due to epidemic.*

***B:*** *1= Shortage of protective supplies; 2= exhausted medical staff; 3= helpless patient; 4= innocent people;*

*5=been infected by virus; 6= been isolated due to epidemic; 7= unsupported by families.*

***C:*** *1= Shortage of protective supplies; 2= possible infection without isolation; 3= possible infection without*

*protection; 4= been infected by virus; 5= New suspected cases; 6= insufficient cooperation of patients; 7=*

*death after infection; 8= new foci; 9= death number; 10= New suspected cases; 11= disrupted work or study*

*due to epidemic; 12= been isolated due to epidemic.*

***D:*** *1= Shortage of protective supplies; 2= possible infection without isolation; 3= possible infection without*

*protection; 4= irresponsible rumor; 5= insufficient cooperation of patients; 6= insufficient attention of unit; 7=*

*unsupported by families; 8= been isolated due to epidemic.*

**Figure S1** The sources for the negative emotions of frontline medical staff. (A) Source of

anxiety. (B)Source of sadness. (C) Source of fear. (D) Source of anger.


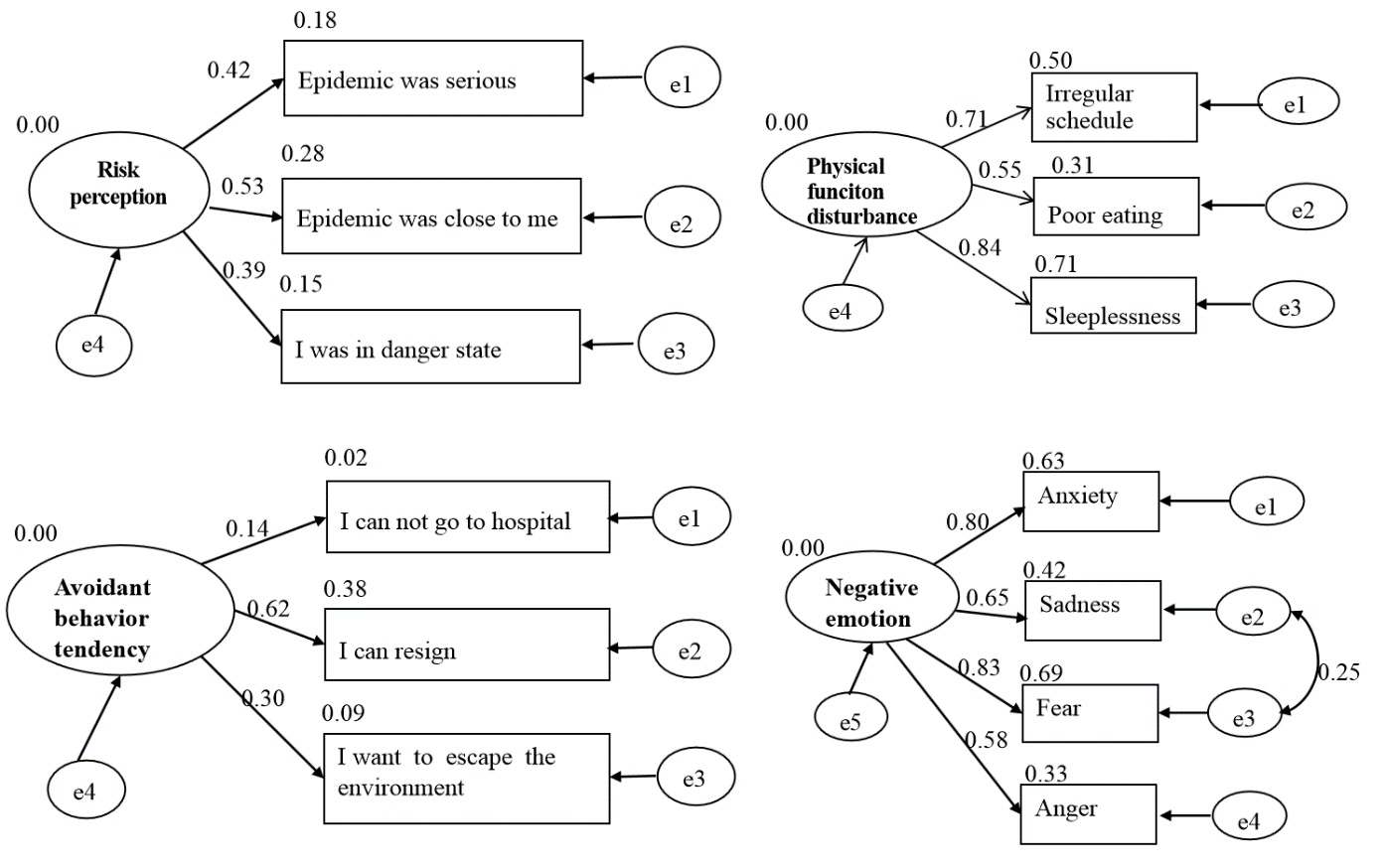


**Figure S2** Confirmatory factor analysis of results
